# Occupational Therapy Professional Identity: Learning From the *Muriel Driver Memorial* Lectures

**DOI:** 10.1177/00084174251327348

**Published:** 2025-03-20

**Authors:** Yasmine Frikha, Andrew Freeman, Nancy Côté

**Keywords:** History, Occupational therapy, Professional association, Professional identity, Association professionnelle, ergothérapie, histoire, identité professionnelle

## Abstract

**Background.** Given the well-documented professional identity challenges experienced by occupational therapists, reinforcing the profession's identity (collective and individual) is crucial for navigating changing environments and optimizing its contribution. The *Muriel Driver Memorial Lectureship* is an important component of the collective identity of the profession in Canada. **Purpose**. A professional identity lens was used to trace the evolution of the profession's collective identity in Canada through this lectureship. **Method.** Using sociological professional identity theory, a documentary longitudinal analysis was conducted on the 43 published lectureship articles (1975–2023), identifying key messages, values, knowledge, and practices. **Findings.** Eight main themes were identified: professional identity, epistemology, axiology, change and leadership, contribution, history, quality, and technology. The analysis revealed an evolving common base of values (occupation, client-centred, social justice) and knowledge (occupation-centred). Persistent challenges included defining theoretical foundations, resisting the biomedical model, and realizing the social vision in practice. The lectures highlighted occupational therapists’ evolving roles and ability to contribute to and lead change. **Conclusion.** The lectures provide insights into the evolution of occupational therapy's collective identity in Canada. Despite ongoing challenges, the contemporary context appears to be increasingly favourable for occupational therapists to practise consistent with the collective identity trends identified.

## Résumé

**Description.** Compte tenu des défis d’identité professionnelle bien documentés que connaissent les ergothérapeutes, le renforcement de l’identité (collective et individuelle) de la profession est crucial pour naviguer dans des environnements changeants et optimiser sa contribution. Le Discours commémoratif Muriel Driver est un élément important de l’identité collective de la profession au Canada. **But.** Le prisme de l’identité professionnelle a été utilisé pour retracer l’évolution de l’identité collective de la profession au Canada par l’entremise de ce discours. **Méthodologie.** En utilisant la théorie sociologique de l’identité professionnelle, une analyse documentaire longitudinale a été menée sur les 43 discours publiés (1975–2023), identifiant les messages clés ainsi que les valeurs, les connaissances et les pratiques transmises. **Résultats.** Huit thèmes principaux ont été identifiés : identité professionnelle, épistémologie, axiologie, changement et leadership, contribution, histoire, qualité et technologie. L’analyse a révélé une base commune de valeurs (occupation, pratique centrée sur la clientèle, justice sociale) et de connaissances (approche axée sur l’occupation) qui évolue. Les défis persistants comprennent la définition des fondements théoriques, la résistance au modèle biomédical et la réalisation de la vision sociale dans la pratique. Les discours mettent en évidence l’évolution des rôles des ergothérapeutes et de leur capacité à contribuer au changement et à en prendre l’initiative. **Conclusion.** Les discours donnent un aperçu de l’évolution de l’identité collective de l’ergothérapie au Canada. Malgré les défis qui persistent, le contexte contemporain semble de plus en plus favourable à ce que les ergothérapeutes puissent exercer leur profession de manière cohérente par rapport aux tendances identifiées en matière d’identité collective.

## Introduction

It is important for occupational therapists and the profession to reflect on therapists’ professional identity in order to act strategically in changing environments and optimize the profession's contribution to healthcare and society (Turner & Knight, 2015). Professional identity refers to those who are, or act as, professionals, which forms the basis for practitioners’ individual identity and the profession's collective identity (Fitzgerald, 2020). These two levels are intertwined because the individual identity draws from the collective identity through the transmission of shared values, knowledge, and practices (Fitzgerald, 2020). This integration occurs through the socialization process resulting from the interaction between an individual and others within a defined context (Dubar, 2015). Multiple challenges that undermine occupational therapists’ identity have been recognized (Mak et al., 2022; Walder et al., 2021). Professional associations are an important component of the occupational therapy profession and thus a means of strengthening therapists’ professional identity (Larouche & Legault, 2003). In this article, we used a professional identity lens, anchored in sociological theories, to trace the evolution of the profession's collective identity in Canada as conveyed by the Canadian Association of Occupational Therapists (CAOT) through the *Muriel Driver Memorial Lectureship*.

A considerable body of literature exists regarding the challenges faced by the occupational therapy profession to unify its identity, notably regarding its ontological foundation (Walder et al., 2021). This challenge primarily concerns the adoption of a unifying perspective centred on occupational science (Turner, 2011; Turner & Knight, 2015) versus a pluralistic approach that promotes the diversity of occupational therapy knowledge, practices and contexts (Guajardo et al., 2015; Mosey, 1985). This profession-level issue may impact practitioners (Mak et al., 2022); therapists have reported difficulties defining what they do with their clients and colleagues, and asserting their place within interprofessional teams (Turner & Knight, 2015). Other investigations described the challenge of providing occupation-centred practice, particularly in practice settings dominated by a biomedical orientation (Walder et al., 2021). Furthermore, the lack of recognition by other team members of occupational therapists’ competencies and roles has presented a challenge for their professional identity (Sauvageau et al., 2017). Mak et al. (2022) scoping study underscored the potential difficulties that occupational therapists might encounter in upholding the profession's philosophy and executing their roles when they perceive a lack of understanding or recognition for their work.

Clarifying the profession's philosophy would help to unify its vision and contribute towards resolving the tensions associated with establishing a single theoretical model, a specific knowledge base, and articulating values, beliefs, and principles in a way that is consistent and understandable for occupational therapists (Hooper & Wood, 2014; Walder et al., 2021). Some initiatives in this regard have been undertaken regarding occupational therapists’ values (Aguilar et al., 2014; Drolet & Désormeaux-Moreau, 2014). Nevertheless, the transmission to, and integration of, the profession's values and knowledge foundation by occupational therapists for the construction of their individual professional identity remain an area of concern. Socialization with peers, through entry-level education at first and subsequently with colleagues, is the main vehicle for this transmission and integration (Dubar, 2015). However, the shift towards integrated care and interprofessional teams also raises identity issues for professionals; one risk is that professional identity is diluted as professionals work with various disciplines and have less intra-professional contact (Best & Williams, 2018). Therefore, it is important to identify other socialization contexts that facilitate interaction between occupational therapists.

Belonging to a professional association constitutes a socialization space for professionals in which the values and knowledge comprising the profession's collective identity are transmitted (Larouche & Legault, 2003). The integration of this identity by professionals such as occupational therapists depends on the clarity of these values and knowledge and their sense of continuity as they evolve over time (Larouche & Legault, 2003). The integration process is also highly context-dependent, as the construction of professional identity is influenced by factors such as organizational environments, interprofessional dynamics, and societal norms and values (Porter & Wilton, 2020). The integration of this identity is reflected in professionals’ role-related actions, as they often define themselves by the work they do (Reay et al., 2017). Professional role identity describes the relationship between the role, that is, the actions that professionals perform, and the identity, which reflects the professionals subjective representation of their role (Ibarra, 1999). Thus, understanding the collective identity requires focus both on the profession's values and knowledge foundations and on their enactment in roles and practices. However, no research has been conducted that specifically examines the role of professional bodies in the development of occupational therapists’ professional identity or how it influences their practice (Mak et al., 2022).

In Canada, the CAOT plays an important role in the dissemination of new knowledge and innovative practices specific to the profession (e.g., through conferences, professional development activities, practice networks). Among CAOT's initiatives include the *Muriel Driver Memorial Lectureship*, which has been awarded annually since 1975 to occupational therapists who have made outstanding achievements in the profession in the areas of research, education and practice. This lectureship is the highest honour in the profession in Canada; the recipients are invited to deliver a lecture to challenge the profession, question its fundamental concepts and practices, and highlight the challenges facing it to foster its development (Finlayson, 2008). This lectureship thus provides an opportunity to trace the values, knowledge and practices, and their evolution over time, of the profession in Canada. Situating the evolution of these core elements over time can provide valuable insights into the historical contexts in which the profession has developed. The purpose of this article is to capture the overall trends in the collective identity conveyed by this lectureship.

## Methods

### Design

With rare exception, each lecture was subsequently published in the *Canadian Journal of Occupational Therapy* (CJOT). We conducted a documentary analysis of these articles, that is, an analytic procedure for reviewing documents that entail finding, selecting, appraising and synthesizing data (Bowen, 2009). This approach is appropriate for analyzing historical facts and documents in the context in which they were written (Saetta, 2016). Given that the lectures were delivered over nearly 50 years, our analysis constituted a longitudinal exploration to better understand how the content of these lectures evolved over time alongside contextual changes.

We recognize two potential biases in this research design. Regarding subjectivity bias, authors are inherently influenced by their own experiences and interests, which inevitably shape the content of the discourse. Selection bias refers in our case to the choice of the awarded lecturer, as their perspectives may align with the policies or interests of the organization (Bowen, 2009).

### Constitution of the Corpus

The lectureship began in 1975; with minor exceptions, lectures have been delivered once a year. No articles were published for the years 1978, 1995, 1996, 1998, and 2020; therefore, a total of 43 articles were included in the analysis.

### Data Extraction

Because the lectures covered a variety of topics, it was necessary to capture the essence of each presentation to permit systematic analysis. We aimed to establish a rigorous process to capture the essence of the messages conveyed by each author without diluting or distorting them, yet with sufficient detail for analysis. Following experimentation, it became clear that the best way to achieve this goal was to define the main theme and three key messages for each article, thus achieving a balance between capturing the essence of the lecture and maintaining conciseness. The first two authors conducted their extraction and synthesis independently. Subsequently, a discussion took place to reach a consensus. A fourth message could be identified on an exceptional basis if required.

### Analysis

Document analysis involves an iterative process combining thematic analysis - identifying patterns in the data, with themes serving as analytical categories—and content analysis, which organizes information into categories (Bowen, 2009). We first analyzed the synthesized data thematically with predefined codes based on the concepts regarding professional identity presented in the previous section; that is, collective identity is formed from a common base of values and knowledge, and is reflected in therapists’ roles and practices (Fitzgerald, 2020; Larouche & Legault, 2003). Consistent with these concepts, themes should highlight values, knowledges, roles or practices emerging from the lectures. Parallel with this thematic analysis, we used content analysis to ensure a precise understanding of each author's key messages, which allowed us to identify insights for the profession that do not refer explicitly to professional identity. More than one theme could be identified within a lecture. We used a reflexive approach, which involved considering how our personal values, experiences, and interests might influence our interpretation (Morgan, 2022). Ongoing discussions allowed us to remain respectful of the lecturers’ original intent while being attentive to the meaning conveyed in the discourses (Bowen, 2009).

Data regarding each theme often emerged from several lectures delivered at different times. Thus, we also conducted a longitudinal analysis of the themes to capture how the values, knowledges, roles, practices, or challenges evolved over time. From a professional identity perspective, it is useful to identify elements of continuity or rupture because it affects the solidity of the professional identity (Dubar, 2015).

## Findings

We identified the following eight main themes across the published lectures: professional identity, epistemology, axiology, change and leadership, contribution, history, quality, and technology. Table 1 provides the results of our categorization and each lecture's key messages.

**Table 1.** Authors, Main Theme and Principal Messages of Muriel Driver Memorial Lectures.

Year	Author, Main Theme, Principal messages	Theme
1975	**Bassett: Occupational therapy’s positioning within the health care system and how its role can be enhanced and effectively advanced** • Occupational therapists have traditionally responded to the unmet needs in the hospital thanks to their adaptive skill and determination, leading to greater recognition. • However, for various reasons, therapists have been reluctant to advocate for the profession, which has increased its vulnerability. • It is important to advance the profession’s knowledge and expertise and clearly explain its contribution, which can potentially include several new roles (e.g., social rehabilitation, intervention for high-risk babies, employment adjustment) within and outside the traditional biomedical services. • Nevertheless, certain important barriers (e.g., ressources priorities) remain that require concerted efforts to overcome.	Change & Leadership • Contribution
1976	**Hood: The importance of maintaining competence throughout the career** • Occupational therapists must maintain their competencies throughout their careers to provide the best possible service quality. • Various mechanisms exist to support competency maintenance (e.g., credentialing, peer review, self-evaluation). • Continuing education is essential for supporting evolving occupational therapy roles, including the negotiation of these roles.	• Quality
1977	**O’Shea: The role of professional interaction in professional identity construction** • Historically, the professional image of occupational therapy has been shaped more by the expectations and demands of others. • Interactional theory is useful for conceptualizing occupational therapists’ development and projection of their professional identity. • Occupational therapists must accept the responsibility of creating a professional identity that is congruent with their own definition of occupational therapy and become independent; they must project a professional image that will convince others of their capabilities and effectiveness.	Contribution • Epistemology • Professional identity
1979	**Gilewich: The importance of, challenges facing, and development of occupational therapy managers** • Occupational therapy managers are faced with various challenges (e.g., be accountable, maintain the universal perspective of occupational therapy, communicate effectively, continuing education). • These managers require several competencies, opportunities for the development of which must be available and used. • Occupational therapists need to be educated in management theory, including in their entry-level formation.	• Change & Leadership
1980	**Bell: Role theory and role negotiation skills are important components of occupational therapist competencies** • Excessive role-taking (understanding and adopting the roles of others) might not always be professionally appropriate; conversely, excessive role-making (planned creative changes) might be too insular and fail to align with the roles of others. • Following the development of a strong professional identity and positive professional image, role negotiation skills should be employed to further advance and define professional roles. • Occupational therapists’ skills at negotiating roles effectively with health care facilities, universities and governments will be the focus of professional action in the next decade.	Contribution • Epistemology • Professional identity
1981	**Robinson: Early development of occupational therapy and CAOT in Canada** • Recognition that developing the occupational therapy profession required the establishment of a scientific underpinning. • Clarity about the underlying premise of occupational therapy: through mind and hand to health. • Describing the establishment of entry-level education and some early occupational therapy roles (e.g., war-injured, tuberculosis sanatoria; home care).	Axiology • Epistemology • History
1982	**Judd: A historical perspective about occupational therapy using an older adults services lens** • The occupational therapy profession must constantly evolve and demonstrate adaptability linked to its holistic perspective. • Occupational therapists’ practice is based on a clear set of values (e.g., related to older adults) and related competencies. • These competencies translate into a range of roles in different settings that need to be developed (e.g., within institutions, community-based). • There is a need to better prepare occupational therapists for the challenges in gerontology and to modify their attitudes toward older adults.	Axiology • Contribution • History
1983	**Forget: Applying a systems paradigm to the occupational therapy evaluation of older adults** • The occupational therapy and biomedical paradigms are incompatible. • There is a strong intrinsic link between occupational therapy and the systems paradigm (e.g., open, complex and multiple systems in a dynamic relationship with the environment). • This paradigm can be well illustrated by occupational therapy practice with older adults.	• Epistemology
1984	**Saunders: The importance of quality assurance and its implications for occupational therapy** • Assuring the quality, equity and accessibility of occupational therapy services is essential. • Professional accountability is important: occupational therapists should use rigorous standards, including outcome measures, for evaluating quality. • All occupational therapists have a role to play in, and advocate for, the application of quality standards in every aspect of professional practice.	Change & Leadership • Quality
1985	**Brintnell: Career planning in occupational therapy** (*I want up, not out*) • Given historical patterns and current demographic trends for rehabilitation services and health system costs, it is important for occupational therapists to take responsibility (e.g., seek advanced credentials) for influencing the career tracks and opportunities that are available. • This evolution must consider certain barriers (e.g., gender discrimination, power, overwork), some of which are linked with being a female-dominated profession. • Occupational therapists need to professionally assert themselves and develop the necessary competencies (e.g., mentoring, networking, positive power behaviors).	• Change & Leadership
1986	**Gill: Commitment to the profession** • Continuing education is an essential manifestation of professional commitment given rapidly advancing knowledge and the need to demonstrate cost effectiveness • A strong commitment to voluntarism is vital for the advancement of a young and dynamic profession. • According to the findings of a survey conducted with members of the occupational therapy profession in Canada, personal satisfaction is the main reason for involvement in the professional associations; time is the biggest deterrent.	Contribution • Quality
1987	**Stan: Acting strategically to maximize occupational therapy’s impact** • It is important to understand the multiple influences (e.g., resource constraints; consumer demand for value; moving away from biomedical model; emerging roles for women) on occupational therapy to move forward. • Certain underlying principles should guide the profession going forward: service to others, holistic perspective, occupation as the core, collaboration. • To be more strategically positioned within the changing health care context, occupational therapists can: take on more roles (e.g., advocacy, community consultant, educator, supervisor), pursue lifelong learning, participate in research (e.g., generate economic evidence, new models of organization) and clearly define the occupational therapy role.	Axiology • Change & Leadership • Contribution
1988	**Baptiste: Chronic pain as a lens for exploring activity and culture** • The occupational therapy profession must re-examine the relevance of the foundational construct of practice *activity/occupation* to ensure relevance to clients’ cultural needs. • Insufficient consideration of culture in health results from the employment of very narrow biomedical models in clinical practice. • A key ingredient for effective health and wellness includes consideration of the spiritual resources of human beings, by adopting a meaning-centred approach.	Axiology • Epistemology
1989	**Tompson: Increasing the visibility and viability of the profession** • The visibility of the occupational therapy profession is reduced by role definition challenges and devaluing, and the diversity of occupational therapy practice; however, public education and the quality of care delivered can reverse this trend. • There are 4 approaches to consider in promoting the profession: (1) self-image; (2) projection of this image through appearance and communication; (3) clarification of roles in relation to societal needs; and (4) active promotion of the profession at the collective and individual levels. • Promoting the profession ensures its viability, that is, its ability to develop and flourish.	• Contribution
1990	**Carswell-Opzoomer: A logical fit between occupational therapy and determinants of health paradigm** • The occupational therapy profession needs to be committed to certain values (e.g., meaningful life for everyone) that underpin its belief in the use of activity for the healthfulness of the human spirit. • The determinants of health paradigm, based on certain values (e.g., social and economic equity, self-esteem, empowerment) is closely aligned with the model of occupational performance. • The profession should consistently adhere to this paradigm in its promotion of occupational performance.	Axiology • Epistemology
1991	**Law: The environment: a focus for occupational therapy** • Environmental barriers infringe the rights of individuals with disabilities. • It is essential that occupational therapists understand restrictive environmental elements, and the key concepts (e.g., occupation, classification of environments/environmental factors) underlying their response, and the integration of environmental concerns into their practice. • Occupational therapy practice must respect certain principles for environmental intervention (goals, definition of need, built environment, adjusted power relations, use of technology, programme planning and related roles) and address the social and political dimensions of the environment.	Axiology • Epistemology
1992	**Polatajko: Naming and framing occupational therapy** • It is essential to name and frame occupational therapy in positive and powerful terms in order to gain wide recognition. • This naming and framing is based on a clear set of values (e.g., that humans are occupational beings), from which clearly emerges the Enablement Model. • The priority of occupational therapy is to enable occupational competence guided by an understanding of the individual, the environment, and their interaction in determining occupational competence.	Axiology • Epistemology
1993	**Townsend: Occupational therapy’s social vision** • Occupational therapy’s foundational values and philosophy (e.g., client-centred practice, therapeutic use of occupation) are consistent with the essential features of social justice (e.g., interdependence, the nature of power, contextual influences, change, collaboration, spirituality, risk, and global inclusiveness). • However, the profession’s vision has been narrowed to comply with dominant community, managerial and medical approaches to disability and ageing. • Occupational therapy's social vision has important implications for how practice is realized (e.g., environmental- rather than individual-level practice, critical analysis, advocacy, collaboration consistent with horizonal power relationships).	Axiology • Contribution
1994	**Picard-Greffe: Embracing competence and ethics** • Various challenges associated with the macro-level environment have emerged for the occupational therapy profession (e.g., aging population; technological progress; information revolution; environmental preoccupations; citizens’ expectations; consumerism; resource pressures). • More than ever, these challenges highlight the need to consider ethics within the profession to make choices in accordance with human and social values (e.g., clients’ rights, client-centred practice). • Occupational therapists must be able to act strategically within the practice environment, which necessitates, among other elements, a good understanding of self.	Axiology • Contribution
1997	**Gage: Synergistic health care teams situated within interdependence** • Within programme approaches, building synergistic teams with the client and the other professionals, which require an interdependence mindset, is essential to meet clients’ needs. • Several ingredients are required, including a clear process (building the foundation; establishing a common vision; nurturing the ongoing relationship). • Occupational therapists need to foster change in their teams and be a role model for collaborative teams.	Change • & Leadership • Epistemology
1999	**Westmorland: Risk taking as an antidote to diffidence** • The environmental factors restricting the occupational therapy profession highlight the need for therapists to take risks to remain consistent with their values, and to advance the profession (e.g., promoting the contribution to government). • Risk-taking is a competency to be developed and can benefit therapists who take risks as well as the profession. • Risk-taking can be carried out at the system and individual level, and can take a variety of forms (e.g., team leader roles, facilitate programme changes, introduce research-based approaches).	Axiology • Contribution • Change & Leadership
2000	**McColl: Spirit, occupation and disability** • The occupational therapy profession has traditionally been interested in two specific aspects of spirit: spirituality among people with disabilities and spirituality experienced through occupation; these considerations have certain limits. • Despite being incorporated in the Canadian occupational therapy model, many therapists are not comfortable with the notion of spirituality, which can be a source of distress and guilt. • Some new perspectives are proposed, as are various implications for occupational therapists.	• Epistemology
2001	**Fearing: Change: Creating our own reality** • Change, which is a permanent characteristic of occupational therapists’ practice environments, has both positive and negative impacts at the personal, professional, and organizational levels. • Occupational therapists, who can choose how they respond to imposed change, must understand change, and develop the competencies for responding strategically (e.g., advocacy, negotiation). • By naming, and advocating for, their values and building connections in the workplace, they can lead change to help create contexts that facilitate client participation and well-being at work.	• Change & Leadership
2002	**Thibeault: In praise of dissidence** • Occupational therapists’ core goal is to heal through meaning (motivation, engagement, lasting healing). • The profession, with occupation as its most fundamental tool, should focus more on mindfulness and less on conformity, thus resisting social pressure and challenging commonly held views. • Occupational therapy, as a dissident profession, should foster clients’ true maturity, complete their natural sequence of development, and show concern for social justice.	Axiology • Contribution
2003	**Friedland: Influence of crafts on the development of occupational therapy in Canada from 1890 to 1930** • Three foundational ideas underlie the use of crafts in occupational therapy: (1) health and art are connected; (2) learning by doing; (3) crafts are essential to providing life necessities. • Three social movements are linked with the use of crafts in occupational therapy as a medium for working with those who were in some ways disadvantaged: (1) Arts and Crafts movement; (2) Settlement House movement; (3) Mental hygiene movement. • Occupational therapists need to reconnect with their social vision and work with those who are disadvantaged or marginalized (e.g., poor, mentally ill, homeless) to decrease their dependency and improve the quality of their lives by engaging in meaningful occupations.	Axiology • History
2004	**Backman: Occupational balance: exploring the relationships among daily occupations and their influence on well-being** • Despite occupational balance being one of the original concepts underlying occupational therapy, it remains an abstract and evolving concept that lacks clear definition. • Further research is required to test the hypothesis that occupational balance makes a significant contribution to health, well-being and life satisfaction. • There is a need to move beyond the categorization of self care, productivity and leisure when measuring occupational balance, and grapple with the multiple occupations that comprise a person's day, the characteristics of the occupations and the individual engaged in them, and how that influences health.	• Epistemology
2005	**Desrosiers: Participation and occupation** • Two conceptualisations of the disablement process, the ICF and DCP, provide some consensus about participation, but also some variability (e.g., which life domains may conceptually be included in participation; whether the activity and participation dimensions of the ICF model should be distinct). • Although considerable work remains to be done, the evolution of these models has led to advances in understanding regarding the assessment, predictors, and evolution of participation • Occupational therapists are well positioned to influence other members of the health care team to put participation front and centre in the ultimate aim of the interventions.	Change & Leadership • Epistemology
2006	**Miller **P**olgar: Assistive technology as an enabler to occupation** • To maximize the usefulness of assistive technology for clients, it is essential to consider the meaning assistive technology holds for them as well as their personal preferences when engaging in occupations. • This perspective is congruent with the concepts of client centred practice and within the Canadian Model of Occupational Performance that guide therapists to explore clients’ occupational engagement. • Working within this perspective has implications for the specific role of occupational therapists at different stages in clients’ rehabilitation (e.g., education and information; facilitation; listen and collaborate).	Axiology • Epistemology • Technology
2007	**Egan: Speaking of suffering and occupational therapy** • Although perhaps uncomfortable, it is essential for occupational therapists to acknowledge clients’ suffering. • Numerous occupational therapy interventions exist to alleviate remediable suffering (e.g., enabling occupations, valued self-care occupation, use of adaptation). • Nevertheless, therapists also must avoid the infliction of further suffering, continue the development of equanimity and compassion in the face of irremediable suffering, provide safe spaces for patients to try new ways of being, and reflect on their own values and beliefs.	Axiology • Epistemology
2008	**Krupa: Addressing stigma in mental illness within the profession** • It is essential to be aware of what stigma is, and its impact. • Large scale social issues are perpetuating stigma toward people experiencing mental illness; the occupational therapy profession might also be contributing to it. • Occupational therapists, in their efforts to address stigma, should enlarge the vision of socially-centred occupations, undermine stigma processes occurring in routine clinical encounters, and address structural mechanisms that influence social distancing in occupational therapy.	Axiology • Change & Leadership
2009	**Korner-Bitensky: Occupational therapy as a thread that weaves a lifetime** • Occupational therapy as a career is an intrinsic part of one’s life. • Involvement in this profession is an important source of gratitude and enriched understanding, for example, regarding perspectives on health and the strength to adapt, lessons in daily life, parenting, money or the lack thereof, and illness. • Everyone has a specific personal experience of being an occupational therapist.	• Axiology
2010	**Majnemer: Life balance: importance of leisure to well-being and social engagement** • Life balance, including leisure activities, is vitally important for individuals’ physical and mental health. • Environmental factors play a vital role in influencing participation in leisure activities. • It is important for occupational therapists to use their knowledge and skills to address barriers to participation in leisure activities by, for example, working with individuals, organizations and communities to advocate for policy and practice changes.	Change & Leadership • Epistemology
2011	**Letts: Optimal positioning of occupational therapy: application in primary health care** • The primary focus of occupational therapy is occupation, emerging from its historical roots and its basis in occupational science. • Despite the universal experience of occupation, the profession must consider the risks and opportunities of exploring new roles and maintaining existing services. • To position occupational therapy optimally, three key questions must be asked: (1) how proximal is occupation in the role? (2) How strong is the evidence? (3) Is the timing right for change? • The underlying principles of primary health care reform are strongly aligned with an optimal occupational therapy contribution.	• Contribution
2012	**Cooper: Reflections on the professionalization of occupational therapy** • The occupational therapy profession comprises several theoretical components: traditional embrace of reductionism; evolving theoretical foundations; occupational science as the central organising tenet; client-centred practice; the role of the environment in understanding clients’ occupational functioning; advocacy. • Despite the professionalization of occupational therapy over the last 50 years, it continues to be faced by both external (e.g., restrictions on scope of practice; competition from other professions; challenges of explaining occupational therapy) and internal (e.g., diffidence; fragmentation; insufficient advocacy competency) challenges. • A shift is required to the external dynamic, the public arena, requiring certain roles: leadership, advocacy services to individuals with chronic conditions; roles associated with addressing determinants of health.	Axiology • Change & Leadership • Epistemology
2013	**Finlayson: Embracing our role as change agents** • Change, and thus also the change agent role, are an intrinsic part of occupational therapy practice. • To be effective, therapists must understand the elements underpinning this role (e.g., communication effectiveness; conflict resolution; insightful; reflective; disciplined; visionary leaders and mobilizers; knowledge integrators and translators; diplomatic interventionists). • This role, which is relevant for all occupational therapists regardless of job title, includes several components (e.g., advocacy, collaboration, enabling competencies).	Change & Leadership • Epistemology
2014	**Dubouloz: Transformative occupational therapy** • Transformation, which is situated at the core of the client-centred approach, is one of the key impacts that occupational therapists have on their clients. • In the context of the various perspectives that exist about transformation, the *Meaning Perspective Transformation Process* is proposed, which includes three phases: (1) trigger, (2) changing, (3) outcome. • Incorporating transformation dimension into occupational therapists’ practice includes various specific actions.	Axiology • Epistemology
2015	**Kirsh: Integration of advocacy and social justice into occupational therapists’ identity** • The dominant individualist perspective overshadows the social and structural roots of occupational injustice. • In becoming more socially and politically active, the profession will face certain challenges: their magnitude; the pressure to conform with particular managerial approaches and a biomedical orientation; the political climate of neoliberalism. • Occupational therapists must embrace the promotion of social change through advocacy in any research or practice role.	Axiology • Change & Leadership • Professional identity
2016	**Gélinas: Collaborative partnerships between practitioners and researchers** • Collaborative partnerships in occupational therapy research can facilitate practitioners’ participation in research activities and better ensure that research initiatives are conducted from the point of view of the practitioners and thus are more relevant to clinical practice. • Key factors for building and sustaining meaningful partnerships include the presence of favourable pre-partnership conditions related to the context and the use of guiding principles focusing on vision, values, trust, communication, power sharing, and interactions. • Many of these factors are consistent with occupational therapy core competency roles and reflect occupational therapy professional values.	Axiology • Quality
2017	**Whalley Hammell: Opportunities for well-being: the right to occupational engagement** • Occupational engagement is fundamentally important to human well being, which is a human right. • Therefore, occupational therapists need to go beyond the biomedical scope and an individual abilities orientation to focus on capabilities (opportunities to do what they have the abilities to do). • Occupational therapy's importance to society will be manifested when it focuses unambiguously on well-being.	Axiology • Contribution • Epistemology
2018	**Liu: Occupational therapy in the 4**^th^** industrial revolution** • Technology has an impact on citizens and must be used in the profession to advance the common good (e.g., address spatial injustice, enhance citizens’ access to services). • Technology innovations will have an important impact on occupational therapists’ knowledge and competencies (e.g., evolving ideas about occupation). • Technology will have an impact on the nature of clients’ challenges and how occupational therapists provide services, including on their assessments and interventions. • Occupational therapy expertise based on critical thinking, creativity, therapeutic use of self, and occupational enablement will be sought by robotics and artificial intelligence experts, which may lead to an expansion of occupational therapy roles (e.g., technology design and implementation)	Contribution • Epistemology • Technology
2021	**Laliberte Rudman: Mobilizing occupation for social transformation** • The occupational therapy profession must address contextual determinants of occupational inequities, which recognizes the political nature of occupation and subsequently new conceptualisations of occupational therapy (e.g., resisting individualized approaches to social problems and inequities, disrupting processes of marginalization and exclusion). • This work necessitates a radical sensibility (e.g., embracing critical optimism; Utopic imagination; generative disruption). • In turn, several competencies are necessary, for example, critical reflexivity and critically-informed practice approaches.	Axiology • Change & Leadership • Epistemology
2021	**Demers: Expanding occupational perspectives with family caregivers** • Occupational therapists should consider caregivers as clients with important occupational needs, goals, and desires that are related to caregiving as well as other facets of their live. • Although caregiving can be burdensome, but it can also create positive effects for the family caregivers, many of which can be identified and understood through a relational lens. • This reasoning is supported by recent research on occupational therapy values and shifts toward an occupational participation model that enhances a collaborative relationship approach. • A collaborative approach can be used with family caregivers to locate and mitigate negative caregiving effects, discover and build on positive effects, and encourage a balance between caregiving and non-caregiving occupations.	Axiology • Epistemology
2022	**Trentham: Occupational (therapy’s) possibilities: A Queer reflection on the tangled threads of oppression and our collective liberation** • Liberation is discussed as a communal process and outcome of untangling, undoing, and reconfiguring systems of dominance that negatively impact health and limit the occupational possibilities of individuals, groups, and communities. • The transformation toward a practice based on anti-oppressive principles and the liberation of occupational therapy’s full potential must be tied to the liberation of the communities supported by occupational therapy. • It is necessary to build within the profession a representative and compassionate professional community to support collective action and to position the celebration of communal achievements as resistance and acts of gratitude.	Axiology • Change & Leadership • Epistemology

### Findings Explicitly Regarding Professional Identity

Three lectures (O'Shea, 1977; Bell, 1980; Kirsh, 2015)^1^ explicitly addressed professional identity, albeit at differing levels of detail. O'Shea conceptualized professional identity using interactionist theory; she emphasized occupational therapists’ responsibility to create their professional identity and the importance of considering interactions with clients and other professionals in strengthening this identity through the exercise of their professional roles. Bell, in introducing role theory, emphasized the need to build a strong professional identity to facilitate the negotiation of professional roles to develop them further; one crucial element of a successful role negotiation is that of finding a balance between role-taking (understanding and adopting the roles of others) and role-making (planned creative changes). Kirsh emphasized the need to integrate advocacy, at political and public levels, and occupational justice in occupational therapists’ professional identity.

### Findings Reflecting the Collective Professional Identity

Most of the lectures incorporated elements regarding axiology and epistemology, providing a comprehensive overview of the foundations of occupational therapy's collective identity while highlighting the key challenges encountered by the profession. Axiology refers to what constitutes the essence of occupational therapy, in particular, the values that guide actions (Drolet, 2014); epistemology refers to occupational therapy knowledge (models, theories or frames of reference used in occupational therapy) (Hooper & Wood, 2014). The three lectures (Robinson, 1981; Judd, 1982; Friedland, 2003) that provided an overview of occupational therapy's history highlighted the origins of the profession's essence and/or values. When specific roles and practices were presented in various lectures, they were always anchored in values and/or knowledge and not presented in isolation.

Regarding practices, two dominant themes emerged: *contribution*, which concerns the development and promotion of the profession's contribution, and *change and leadership*, which focuses on the competencies required to influence change, advocate for clients or communities, and assume leadership roles. The themes *quality* and *technology*, which were addressed less frequently, stand out from the other discourses. A few lectures presented elements relating to the development or maintenance of the quality of services provided by professionals; they involved processes relating to the development or maintenance of competence, (e.g., through entry-level/ongoing training, research) (Hood, 1976; Gill, 1986; Gélinas, 2016). Professional accountability and methods to evaluate the quality of services were also discussed (Saunders, 1984). Two lectures (Miller Polgar, 2006; Liu, 2018) specifically addressed the use of new technologies in occupational therapy. According to Miller Polgar, technology use can be incorporated into client-centred practice and the *Canadian Model of Occupational Therapy*. Liu, for her part, proposed how technology can transform the way occupational therapists conduct their assessments and interventions, as well as the nature of the challenges encountered by clients and communities. In summary, our findings mainly concern axiology, epistemology, contribution, and change and leadership, the details about which are presented in the following sections.

#### Occupation as Core Values and Knowledge: Evolving from Person/Occupation to Occupation/Environment with a Growing Focus on Social Justice

Axiology and epistemology categories highlight the common base of values and knowledge transmitted by the lectures over time. The profession's values are affirmed by various authors, albeit not necessarily operationalized to practice. Three main categories of core values within the profession emerged, being emphasized to varying degrees across the lectures : (1) occupation: the role of occupation in human life [e.g., mind/hand to health (Robinson, 1981), occupation as the core (Stan, 1987; Cooper, 2012), the meaningful life (Carswell Opzoomer, 1990; Friedland, 2003), the therapeutic use of occupation (Townsend, 1993)]; (2) client-centredness (e.g., Townsend, 1993; Picard-Greffe, 1994; Miller Polgar, 2006; Cooper, 2012; Dubouloz, 2014); and (3) social justice [e.g., interdependence, empowerment, equity, rights (Carswell Opzoomer, 1990; Law, 1991; Townsend, 1993; Friedland, 2003; Whalley Hammell, 2017; Laliberte Rudman, 2021)].

Most of the knowledge elements presented in the lectures relate to occupation. Other areas of knowledge, for example, about change (Finlayson, 2013), suffering (Egan, 2007), transformation (Dubouloz, 2014), liberation (Trentham, 2022), and technology (Miller Polgar, 2006; Liu, 2018) were introduced in the context of occupational therapists being called upon to adopt a new vision of their profession or take on new roles.

Nevertheless, how knowledge about occupation was discussed evolved over time. The person and the environment were always considered in relation to occupation, rather than as separate entities. Initially, the focus was on the relationship between the person and occupation, for example, considering the meaning of occupations for the individual [meaning-centred approach (Baptiste, 1988; Dubouloz, 2014); spirituality (McColl, 2000)], or occupational balance from an individual well-being perspective (Backman, 2004; Majnemer, 2010). Subsequently, occupation became increasingly intertwined with the physical, social, and political dimensions of the environment (Law, 1991; Townsend, 1993; Kirsh, 2015; Whalley Hammell, 2017; Laliberte Rudman, 2021; Demers, 2022).

#### In the Beginning: the Challenge of Structuring the Foundation of Collective Identity Around Occupation

To better understand the evolution of this knowledge and the realization of the values in practice over time, it is relevant to examine the major challenges identified in the lectures to define and enact this collective identity in practice. The first challenge concerned that of defining the profession's theoretical foundations. Robinson (1981) emphasized the importance of establishing a scientific underpinning. Subsequently, several authors focused on defining knowledge specific to the profession. Theoretical models in occupational therapy were discussed by Carswell-Opzoomer (1990) (occupational performance model) and Polatajko (1992) (enabling model). In 2012, Cooper asserted that the profession had solidified its theoretical foundations, suggesting that this challenge had been resolved.

#### From Past to Present: the Ongoing Struggle to Distance Occupational Therapy's Identity from the Biomedical Model

The second major challenge for the profession was that of resisting the biomedical model, a concern raised in 1983 by Forget and subsequently in 1988 by Baptiste. In 1990, Carswell-Opzoomer proposed adhering instead to the determinants of health paradigm due to its alignment with the occupational performance model. In 2004, Backman focused attention on the *International Classification of Functioning, Disability, and Health* and occupational participation, showing how these two conceptualisations could converge. Subsequently, the debate around resisting the biomedical model subsided, only to reappear in 2015 with Kirsh and again in 2017 with Whalley Hammell, which suggests that this challenge had persisted in the profession. Beyond these specific lectures, occupational therapy models were rarely contextualized in relation to paradigms outside the profession.

#### The gap Between Collective Identity and Practice: Enacting the Social Vision and Embracing a Collective Approach

The third challenge involves realizing the social vision of occupational therapy by embracing collective rather than individual approaches. Although rooted in the profession's core values, translating this social vision into practice seems to have been an ongoing challenge. In 1991, Law introduced the issue of the social and political environments, and was the first to mention individual rights. Townsend (1993) articulated a social vision for occupational therapy, Krupa (2008) addressed stigma and socially-centred occupations, and Kirsh (2015) emphasized the importance of being more socially and politically responsible and to go beyond individualist perspectives. In 2017, Whalley Hammell asserted the importance of occupational justice, emphasizing the need to move toward community and population-based approaches; it is crucial to address this challenge to expand occupational therapy's contribution to society. Two subsequent lectures (Laliberte Rudman, 2021, Trentham, 2022) were consistent with this same desire to contribute to greater occupational justice, although their specific orientations differ somewhat.

#### Contribution, Change and Leadership: Shaping Professional Identity and Responding to Society's Needs

The two themes of *contribution*, and *change and leadership*, addressed the profession's evolution and occupational therapists’ roles, although each theme was approached somewhat differently over time. Most of the lectures that addressed the development of the profession's contribution preceded the 2000s. This development was approached from the perspective of the importance of clearly defining occupational therapists’ professional identity (O'Shea, 1977) and their role (Stan, 1987; Tompson, 1989) including the development of new roles in different settings (Bassett, 1975; Judd, 1982; Stan, 1987; Westmorland, 1999). Bell (1980) emphasized the need to develop role negotiation skills to better define actual and future roles. Role development involves considering the influence of the context, and positioning oneself among the various influences, that is, either in a position of resistance against dominant medical and managerial influences (Townsend, 1993; Westmorland, 1999; Thibeault, 2002; Kirsh, 2015; Whalley Hammell, 2017) or in a more nuanced position, based on a thorough understanding of the context and strategic action (Tompson, 1989; Picard-Greffe, 1994; Letts, 2011; Liu, 2018).

Among the new roles identified as needing to be developed was advocacy; first noted by Basset (1975). The discussion about this role was often associated with change and the change agent role. Fearing (2001) was the first to focus specifically on change and how therapists can respond to it; however, she did not explicitly define the change agent role nor the associated competencies. Several other lectures emphasized the importance of adopting an advocacy role or demonstrating leadership to enhance client well-being and participation; this advocacy could involve influencing interprofessional team members (Gage, 1997; Fearing, 2001; Desrosiers, 2005) or advocating for policy or structural changes (Krupa, 2008; Majnemer, 2010). Cooper's (2012) lecture appeared to mark a turning point in the discourse regarding advocacy, emphasizing the need to develop competencies to influence change, with a focus on advancing clients’ and communities’ needs rather than those of the profession. Finlayson (2013) subsequently emphasized the importance of taking on the change agent role, and offered a conceptualization of this role and its underpinning elements. Her argument emphasised that this role is relevant to all occupational therapists, independently of a formal leadership position, as opposed to Gilewich's (1979) and Brintnell's (1985) proposals in which advocacy was associated more with a managerial position. Judd (1982), Krupa (2008), and Trentham (2022) encouraged occupational therapists to reflect on their own attitudes, suggesting that change begins with them. Kirsh (2015) also asserted the importance of integrating advocacy into occupational therapists’ identity.

## Discussion

This article's purpose is to capture the overall trends in the collective identity of Canadian occupational therapists as conveyed by the *Muriel Driver Memorial* lectures. It is important to acknowledge that apart from O'Shea (1977), Bell (1980) and Kirsh (2015), the speakers did not explicitly discuss occupational therapists’ professional identity. This finding is interesting given that professional identity challenges have been discussed within the profession over many years (Mosey, 1985; Walder et al., 2021).

Each speaker's presentation presumably emerged from their professional and academic work and experience, and reflected their analysis of the issues facing the profession during that era. Therefore, we recognize the importance of not attributing retrospective judgements to the presenters’ orientations. Rather, our longitudinal analysis allowed us to recontextualize the different values, knowledge and practices that were conveyed, and to understand what these might tell us about the collective professional identity of Canadian occupational therapists.

Our analysis revealed the contextual and historical trends that have accompanied the profession's evolution. The only two lectures that dealt explicitly with the construction of professional identity were delivered relatively early (O'Shea, 1977; Bell, 1980), and at a time when the professionalization of occupational therapy was rapidly evolving. Other contextual trends include the paradigm shifts accompanied by new theoretical models both within and outside the profession. For example, Forget's 1983 lecture was a continuation of the 1970s movements that questioned the biomedical paradigm as the basis of occupational therapy (Kielhofner, 1978; Reilly, 1971). This lecture followed the publication in 1980 of Kielhofner's *Model of Human Occupation* (Kielhofner, 1980a, 1980b; Kielhofner & Burke, 1980) and came shortly before the publication of the *Guidelines for the Client-centred Practice of Occupational Therapy* (Department of National Health and Welfare & Canadian Association of Occupational Therapists, 1983), which introduced the *Occupational Performance Model*. Polatajko (1992) extended this approach, following the publication of the *Canadian Occupational Performance Measure* in 1990 (Law et al., 1990). Desrosiers’ (2005) conference followed the 2001 publication of the World Health Organization's *International Classification of Functioning, Disability and Health* (World Health Organization, 2001) and the *Disability Creation Process* (Fougeyrollas et al., 1998). Letts’ (2011) and Cooper's (2012) lectures demonstrated the profession's recent evolution and development, as well as the consolidation of its occupation-centred theoretical foundations. Finlayson's (2013) lecture was a continuation of this evolution, following the publication in 2007 of the book *Enabling occupation II: Advancing an occupational therapy vision for health, well-being, & justice through occupation* (Townsend & Polatajko, 2007) and the second edition of the *Profile of Practice of Occupational Therapists in Canada* (Canadian Association of Occupational Therapists, 2012), both of these publications affirming the role of occupational therapists as change agents. Demers’ lecture (2022) was delivered close to the publication of the new *Canadian Model of Occupational Participation* (Egan & Restall, 2022), with its emphasis on collaborative approaches.

This evolution was also reflected in Canadian policies. For example, Carswell-Opzoomer's lecture (1990) took place in the wake of the 1986 adoption of the *Ottawa Charter for Health Promotion* (World Health Organization, 1986), and was part of important conversations about the healthcare system in Canada, which took into account the social determinants of health. In another example, Krupa's (2008) conference took place in a political context marked by growing mobilization for mental health in Canada.

The social context, notably the recognition of social inequalities and cultural diversity, appears to have played a role in the evolution of occupational science, as evidenced by Kirsh's (2015) and Laliberte Rudman's (2021) lectures. Kirsh explored the evolution of occupational science through notions of occupational justice and social, institutional and political contexts. Laliberte Rudman's (2021) messages were communicated in a socio-historical context marked by a growing collective awareness of social inequalities and diversity, reflected in social movements such as *Me Too*, *Black Lives Matter*, and the COVID-19 pandemic, which further exposed social and health disparities. Finally, technological advances have also shaped the evolution - and continue to influence - the profession, as highlighted by Miller Polgar's (2006) and Liu's (2018) lectures.

This historical perspective on contextual trends highlights their influence on the definition of values, knowledge and practices in occupational therapy. Occupational therapists’ professional identity appears to be intrinsically linked to the social, political and technological contexts, and has evolved in parallel with these transformations. Nevertheless, our analysis also suggests that despite this evolution, a common base of values and knowledge persists. This base makes it possible to define the contours of a collective professional identity centred on occupation, which designates both a set of values and a body of knowledge rooted in humanist values and social justice. However, the lectures have not debated what specific knowledge or values should comprise the collective identity, in contrast to the ongoing discussions in the literature regarding the recognition of a plurality or a structuralist view of knowledge within occupational therapy identity (Hooper & Wood, 2002; Guajardo et al., 2015). The lectures appear to align on a foundational body of knowledge based solely on occupation, advocating for its evolution in response to changing contexts. While this finding may contrast with the literature's portrayal of the complexities involved in defining professional identity at both individual and collective levels, it offers a potential strategy for helping therapists define and assert their professional identity around occupation in practice. Concurrently, the lectures highlight the ongoing challenges therapists face in aligning their practice with this identity. The dominant biomedical paradigm represents a major obstacle to actualizing professional values in daily practice; this challenge of delivering occupation-centered practice is well documented (Walder et al., 2021). This difficulty in integrating professional values into everyday practice seems to reflect a gap between the collective identity of the profession and practitioners’ contextual realities.

Nevertheless, the contemporary context seems increasingly favourable for occupational therapists to practise consistent with these fundamental values, thus reducing this gap. As Carswell-Opzoomer (1990) noted, occupational therapists are invited to participate in these contextual developments so that they can create a healthcare system compatible with the foundations of their profession, and thus resolve their identity challenges. Indeed, change is at the heart of occupational therapists’ professional identity and pursues a dual objective: to transform social structures and the healthcare system to improve the well-being of the population, to use Whalley Hammell's (2017) words, and to create favourable conditions for occupational therapists to develop professionally and act consistent with their values. In this sense, it explains why the question of developing the contribution of occupational therapists is intimately linked to roles of change, leadership and advocacy. This point is particularly relevant when analyzed through an identity lens to better understand its implications for professional identity. Recognition by others is essential for strengthening professional identity and alleviating identity tensions (Dubar, 2015). However, this recognition is only truly effective when the recognition bestowed by others is perceived as legitimate in terms of one's own value system (Dubar, 2015). In the past, occupational therapists collectively sought to adjust to the dominant biomedical paradigm to facilitate their professionalization and acquire legitimacy (Turner, 2011). Since Cooper's lecture (2012), the focus has shifted to equipping occupational therapists to influence others to understand their value system, which would enable them to receive the recognition they deem legitimate and thus strengthen their professional identity.

Another implication for professional identity lies in the identity construction of occupational therapists individually. While collective professional identity is shaped by broader social and professional trends, the construction of individual professional identity occurs on a smaller scale. It lies in occupational therapists’ ability to navigate the influences present in their immediate practice contexts through the everyday interactions they have with their clients, interprofessional team members, and other stakeholders (Dubar, 2015). These interactions are shaped by the specific organizational contexts in which therapists operate (e.g., hospitals, rehabilitation centres) where the biomedical model often dominates. O'Shea (1977) emphasized this interactionist perspective in identity construction and invited occupational therapists to consider the role of others, particularly clients, in it. This multiplicity of influences can create tensions when the values and expectations of the profession are at odds with those of these other stakeholders, and can weaken identity construction (Dubar, 2015). In this sense, to achieve a sense of identity cohesion, occupational therapists must develop strategies that will enable them to negotiate these different tensions. Developing critical analysis and reflexivity skills is crucial to resolving the tensions exerted by the external context so that occupational therapists can position themselves strategically (Mackey, 2014).

Most lectures focused on positioning the profession in a broader societal context and developing new roles. It will be interesting to see if future lectures focus more on the immediate contexts of professionals, to equip occupational therapists to analyze the opportunities and constraints present in their environment. In addition, it might be relevant to target the development of negotiation skills to position oneself strategically. Indeed, understanding the context, and collaborating and negotiating with stakeholders are among the competencies that permit occupational therapists to successfully influence change (Picotin et al., 2021).

### Strengths and Limitations

Examining the *Muriel Driver Memorial* lectures over time provides a valuable longitudinal perspective on the evolution of the occupational therapy profession's collective identity in Canada. While very few lectures provided direct insights about professional identity, we situated our analysis in a sociological theory on professional identity construction to better achieve a rigorous analysis. Nevertheless, it is possible that our own research on professional identity and professionalism might have influenced our interpretations. Consistent with our observation that professional identity construction is highly influenced by contextual elements, our findings regarding Canadian occupational therapists’ professional identity might limit the findings’ transferability to other jurisdictions. Nevertheless, the competencies required by occupational therapists to analyze and effectively navigate contextual influences could well be broadly applicable.

## Conclusion

The *Muriel Driver Memorial* lectures provide a rich longitudinal perspective on the collective identify of the occupational therapy profession in Canada, and the associated possibility of influencing therapists’ individual professional identity. Notwithstanding the limited explicit discussion of professional identity in the lectures, several themes with clear links to identity emerged via the information about values, knowledge and practices. Despite the juxtaposition of the lectures with historical contextual elements, a common base of values and knowledge persists, that is, a collective professional identity centred on occupation. Although there might be challenges in closing the gap between this collective identity and the identity of individual occupational therapists, the contemporary context appears to be increasingly favourable for therapists to practise consistent with the tenets of this collective identity. Developing occupational therapists’ capacity to critically analyze their practice contexts and negotiate diverse influences might also help occupational therapists to enact their collective identity.

## Key Messages

- The *Muriel Driver Memorial* lectures conveyed a unified and coherent collective identity of occupational therapists, which can help to clarify therapists’ own identity.
- Occupational therapists’ professional identity is shaped significantly by the societal and organizational environments surrounding both their individual practice and the broader profession.
- Being able to effectively navigate the practice context can strengthen occupational therapists’ professional identity and exercise leadership.

## Notes

1. The references here and elsewhere in this Findings section are presented in chronological rather than alphabetical order to help readers see the evolution over time.
